# Integrated Pristine van der Waals Homojunctions for Self‐Powered Image Sensors

**DOI:** 10.1002/adma.202404013

**Published:** 2024-07-18

**Authors:** Yunxia Hu, Jun Wang, Mohsen Tamtaji, Yuan Feng, Tsz Wing Tang, Mohammadreza Amjadian, Ting Kang, Mengyang Xu, Xingyi Shi, Dongxu Zhao, Yongli Mi, Zhengtang Luo, Liang An

**Affiliations:** ^1^ Department of Mechanical Engineering The Hong Kong Polytechnic University Hong Kong 100872 P. R. China; ^2^ Department of Chemical and Biological Engineering William Mong Institute of Nano Science and Technology and Hong Kong Branch of Chinese National Engineering Research Center for Tissue Restoration and Reconstruction The Hong Kong University of Science and Technology Hong Kong 999077 P. R. China; ^3^ Guangdong‐Hong Kong‐Macao Joint Laboratory for Intelligent Micro‐Nano Optoelectronic Technology School of Physics and Optoelectronic Engineering Foshan University Foshan 528000 P. R. China

**Keywords:** 2H‐MoTe_2_, asymmetric thickness, integrated, self‐powered photodetectors, van der Waals homojunctions

## Abstract

Van der Waals junctions hold significant potentials for various applications in multifunctional and low‐power electronics and optoelectronics. The multistep device fabrication process usually introduces lattice mismatch and defects at the junction interfaces, which deteriorate device performance. Here the layer engineering synthesis of van der Waals homojunctions consisting of 2H‐MoTe_2_ with asymmetric thickness to eliminate heterogenous interfaces and thus obtain clean interfaces is reported. Experimental results confirm that the homostructure nature gives rise to the formation of pristine van der Waals junctions, avoiding chemical disorders and defects. The ability to tune the energy bands of 2H‐MoTe_2_ continuously through layer engineering enables the creation of adjustable built‐in electric field at the homojunction boundaries, which leads to the achievement of self‐powered photodetection based on the obtained 2H‐MoTe_2_ films. Furthermore, the successful integration of 2H‐MoTe_2_ homojunctions into an image sensor with 10 × 10 pixels, brings about zero‐power consumption and near‐infrared imaging functions. The pristine van der Waals homojunctions and effective integration strategies shed new insights into the development of large‐scale application for two‐dimensional materials in advanced electronics and optoelectronics.

## Graphical abstract

Pristine van der Waals homojunctions consisting of 2H‐MoTe_2_ layers with asymmetric thickness are constructed to eliminate heterogenous interfaces and obtain clean boundaries. The layer‐engineered energy bands of 2H‐MoTe_2_ layers induce a built‐in electric field at the interface, enabling self‐powered photodetection. Furthermore, a 10 × 10 array based on 2H‐MoTe_2_ homojunction devices realize zero‐power consumption imaging functions.

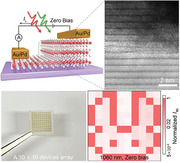

## 1 Introduction

Van der Waals junctions based on two‐dimensional (2D) materials, characterized by their weak interlayer interactions and strong light–mass interaction, exhibit great potential for high‐performance or multifunctional optoelectronics applications.^[^ ^1^, ^2^, ^3^, ^4^ ^]^ The built‐in electrical fields formed at van der Waals junction interfaces facilitate the separation and efficient transfer of photogenerated carriers.^[^ ^5^, ^6^ ^]^ Subsequently, self‐powered photodetectors consisting of van der Waals junctions convert light into electrical signals without external bias, demonstrating promising prospect in zero‐power consumption image sensors.^[^ ^7^, ^8^, ^9^ ^]^ Nowadays, the van der Waals junctions for self‐powered photodetection are mostly composed of two or more types of materials.^[^ ^10^, ^11^, ^12^ ^]^ However, the lattice mismatch in heterojunctions and transfer process during fabrication lead to contamination and defects at the interface including discontinuous band alignments,^[^ ^13^, ^14^ ^]^ strains and traps at interface,^[^ ^15^, ^16^ ^]^ and severe carrier scattering,^[^ ^17^ ^]^ restricting the application of 2D materials in integrated self‐powered optoelectronics.

Van der Waals homojunctions, composed of a single material type, present exceptional boundaries, avoid the rough interfaces in the heterojunctions. These boundaries featured by good uniformity and continuous band alignment serve as high‐quality carrier diffusion channels in optoelectronic devices.^[^ ^18^, ^19^, ^20^ ^]^ Additionally, for thickness‐dependent van der Waals homojunctions, quantum confinement effects of 2D materials induce energy band structures modification between thick and thin regions.^[^ ^21^, ^22^ ^]^ Consequently, the built‐in electric fields at the boundaries in homojunctions result in significant photovoltaic effect, endowing the homojunctions promising potential for the application in the self‐powered photodetectors.^[^ ^23^, ^24^ ^]^ Nevertheless, the current fabrication of homojunctions relies on mechanically exfoliated materials^[^ ^25^, ^26^ ^]^ or synthesized single crystals,^[^ ^23^ ^]^ which suffer from limitations such as uncontrollable thickness, small domain size and irreproducible process. These drawbacks hinder the integration of van der Waals homojunctions into practical image sensors. With the prosperous development of 2D materials in optoelectronics, it is also crucial to develop integrated strategies for homojunctions that are controllable and scalable to wafer size.

Herein, we report the layer engineering of pristine van der Waals homojunctions, which consists of 2H‐MoTe_2_ films through thermally assisted tellurization of patterned molybdenum precursors. The 2H‐MoTe_2_ homojunctions possess asymmetric thickness, avoiding lattice mismatch, chemical bonding, disorder, and defects at the junction boundaries. With the assistance of layer‐dependent energy bands of 2H‐MoTe_2_, a controllable built‐in electric field is formed at the boundaries in the homojunctions, contributing to the construction of a self‐powered photodetector with a broadband ranging from visible (520 nm) to near‐infrared region (1060 nm). Furthermore, an image sensor is fabricated by integrating 2H‐MoTe_2_ homojunctions into 10 × 10 pixels, exhibiting self‐powered imaging functions under near‐infrared illumination. This research presents a facile and feasible approach to facilitate the development of 2D materials for integration into low‐power and wide‐spectrum imaging applications.

## 2 Results and Discussions

### 2.1 Construction of van der Waals Homojunctions

**Figure 1:**
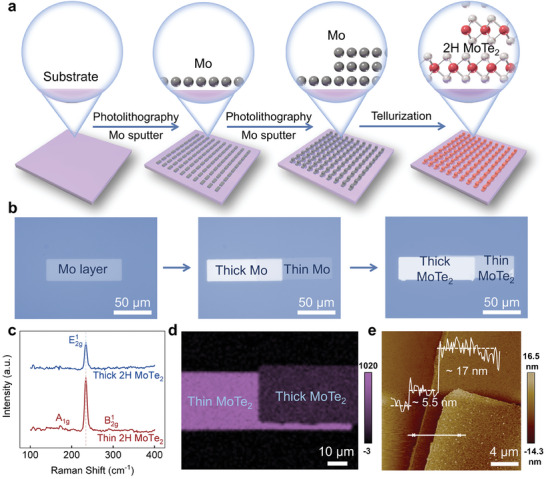
Patterned 2H‐MoTe_2_ homojunction. a) Schematic of the fabrication processes. b) Optical images of patterned Mo layers, layers of different thicknesses, and resulted 2H‐MoTe_2_ homojunctions. c) Raman spectra. d) Raman mapping of E^1^_2g_ mode of the 2H‐MoTe_2_ homojunction with different thicknesses. e) Atomic force microscope (AFM) image of the 2H‐MoTe_2_ homojunction. Inset is the corresponding height profile.

**Figure** **1**a illustrates the fabrication and integration process of pristine van der Waals 2H‐MoTe_2_ homojunctions. A thermally assisted tellurization process is employed based on the molybdenum precursors^[^ ^27^, ^28^ ^]^ with asymmetric thickness. The experimental process and detailed schematic illustrations are shown in Figure 1a and Figure S1 (Supporting Information). First, the molybdenum precursor films are deposited on sapphire substrates using two‐step photolithography and magnetron sputtering processes to form molybdenum patterns with asymmetric thickness. Then the construction of 2H‐MoTe_2_ homojunctions array is achieved via tellurization of the molybdenum patterns using the chemical vapor deposition (CVD) method. During the tellurization process, the molybdenum film first is tellurided into the metallic 1T′‐MoTe_2_ layer, which then transfers into 2H‐MoTe_2_ due to the phase transportation_._^[^ ^29^, ^30^ ^]^ Figure S2a of the Supporting Information shows the optical image of the 1T′‐MoTe_2_ layer with tellurization time of 10 min. The coarse surface of the sample indicates the low crystalline quality of 1T′‐MoTe_2_. Raman spectrum in Figure S2b of the Supporting Information with A_g_ peaks at 108, 128, 162, and 258 cm^−1^ and B_g_ peak at 191 cm^−1^, provides evidence for the successful synthesis of the layered 1T′‐MoTe_2_.^[^ ^31^ ^]^ As the tellurization time increases, the solid‐to‐solid phase transformation from metallic 1T′‐MoTe_2_ to semiconducting 2H‐MoTe_2_ takes place, driving the improvement of crystalline quality and resulting in the disappear of surface particles, as shown in Figure 1b. More details are illustrated in the Experimental Section.

Figure 1b displays the optical images of the molybdenum precursors and 2H‐MoTe_2_ homojunctions with asymmetric thickness. Notably, the 2H‐MoTe_2_ layers exhibit significantly higher contrast compared to the molybdenum layers. Moreover, the brightness of the image increases with an increase in the thickness of the 2H‐MoTe_2_ layer. Besides, the thickness difference of 2H‐MoTe_2_ is determined by Raman spectra and atomic force microscope (AFM) characterizations. As shown in Figure 1c, the Raman spectrum of the thin 2H‐MoTe_2_ layer possesses typical A_1g_ (172 cm^−1^), E^1^_2g_ (234 cm^−1^), and B^1^_2g_ (291 cm^−1^) peaks, while the thick 2H‐MoTe_2_ layer only shows one dominant E^1^_2g_ peak at 234 cm^−1^. The disappearance of the A_1g_ and B^1^_2g_ Raman modes can be attributed to the breaking of translational symmetry caused by the increased thickness of MoTe_2_.^[^ ^21^, ^25^, ^32^ ^]^ Moreover, Figure 1d reveals the spatial distribution of the layer‐engineered 2H‐MoTe_2_ homojunction by the mapping of peak at 234 cm^−1^. All region exhibits a uniform color distribution, with the low‐contrast and high‐contrast areas corresponding to thick and thin layers, respectively. The AFM image and corresponding height profile of 2H‐MoTe_2_ homojunctions in Figure 1e show that the thickness of thin MoTe_2_ and thick MoTe_2_ layer is 5.5 and 22.5 nm, respectively.

### 2.2 Boundaries Characterization of van der Waals Homojunctions

**Figure 2:**
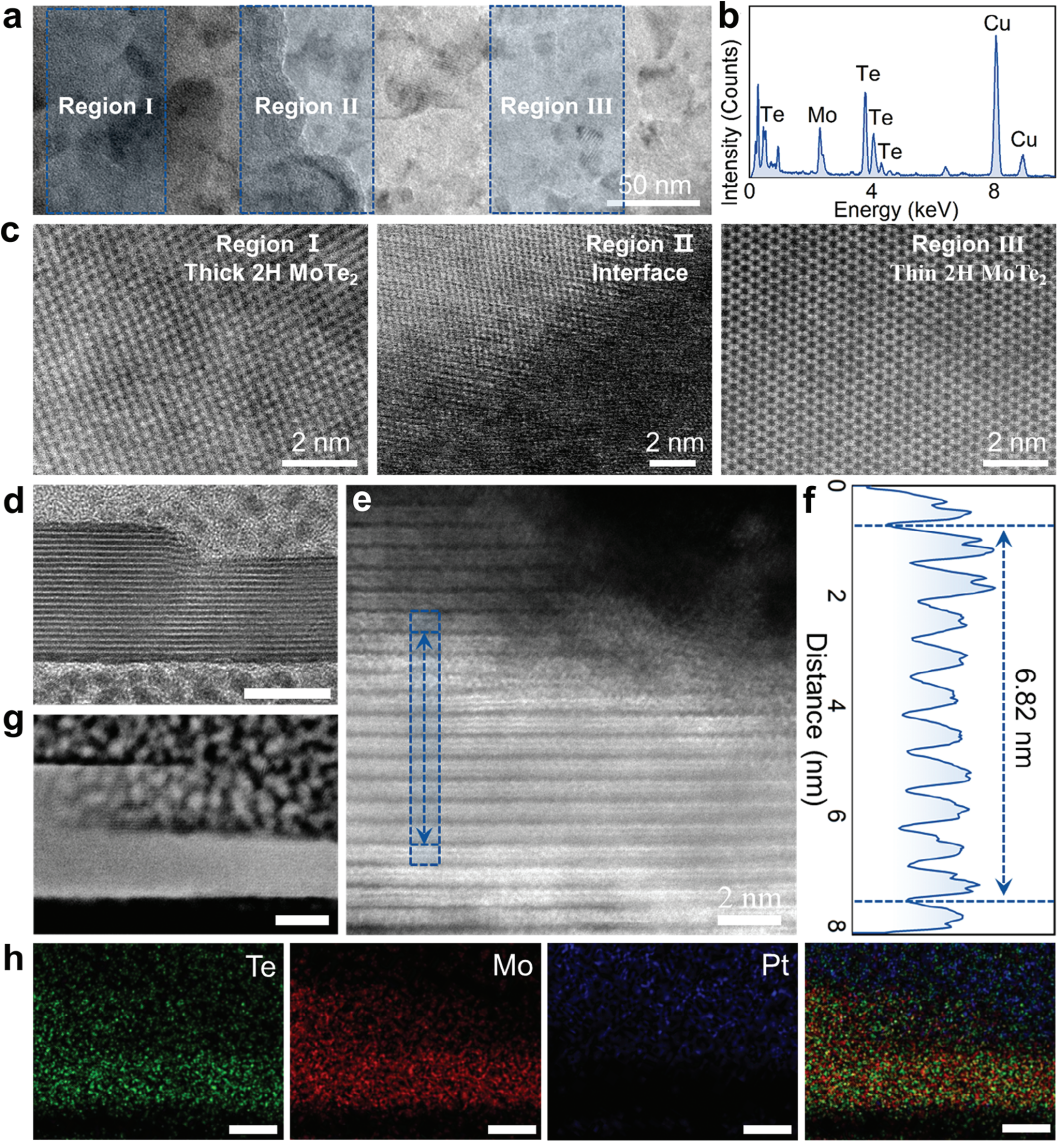
Transmission electron microscope (TEM) images of the 2H‐MoTe_2_ homojunction. a) TEM image at low magnification of the 2H‐MoTe_2_ homojunction structure. b) EDS result of the 2H‐MoTe_2_ layer. c) STEM images of the thick 2H‐MoTe_2_, 2H‐MoTe_2_ homojunction interface, and the thin 2H‐MoTe_2_ regions, respectively. d) Cross‐sectional TEM image of the 2H‐MoTe_2_ homojunction with different thicknesses. The scale bar is 10 nm. e) A large‐scale STEM image of 2H‐MoTe_2_ homojunction atomically sharp interfaces between the thick layer and the thin layer on SiO_2_. f) The line profile extracted from the area of the 2H‐MoTe_2_ in cross‐sectional STEM result marked with a blue rectangle. g,h) EDS elemental mapping images of the 2H‐MoTe_2_ homojunction interface, exhibiting the distribution of Te (green), Mo (red), and Pt (blue) elements, respectively. The scale bar is 10 nm.

This one‐step tellurization method takes advantage of the phase‐transition induced nucleation of 2H‐MoTe_2_, resulting in single crystal nucleation sites at the homojunction boundaries with asymmetric thickness. Therefore, the 2H‐MoTe_2_ homojunction is free from lattice mismatch, strain, and defects, which is defined as the pristine van der Waals homojunction in this work. Transmission electron microscope (TEM) is employed to disclose the morphology of 2H‐MoTe_2_ homojunction, as shown in **Figure** **2**a. Three distinct regions can be identified, including dark thick 2H‐MoTe_2_ region, interface region, and bright thin region. Besides, to investigate the stoichiometry of the grown 2H‐MoTe_2_ layer, energy dispersive X‐ray spectroscopy (EDS) is conducted. The results show that the Te/Mo atomic ratio is 2.02, indicating that there are sufficient Te atoms in the prepared homojunction, which is conducive to the formation of the 2H‐MoTe_2_ phase (Figure 2b). Moreover, the crystal quality of three regions is analyzed at an atomic level using aberration‐corrected scanning transmission electron microscopy (STEM). The STEM images shown in Figure 2c disclose that both the thick and thin MoTe_2_ regions exhibit high crystal quality with a lattice spacing of 0.35 nm, which is associated with (001) plane of 2H‐MoTe_2_.^[^ ^27^, ^33^ ^]^ Different from the distinct atomic stripes with periodic arrangement at regions I and III, the atoms at the homojunction interface are difficult to identify, which may be attributed to the large thickness difference. Additionally, Figure S3 of the Supporting Information illustrates the selected area electron diffraction (SAED) patterns of thick 2H‐MoTe_2_, the homojunction boundaries and thin 2H‐MoTe_2_. The hexagonal patterns observed in Figure S3a,c of the Supporting Information demonstrate the excellent crystallinity of the 2H‐MoTe_2_ phase, while diffraction rings corresponding to polycrystal appear at the homojunction boundaries (Figure S3b, Supporting Information). These findings are consistent with the STEM results, indicating that the thick and thin MoTe_2_ regions are single crystals, and the boundary part is polycrystalline.

Furthermore, high‐resolution TEM is performed to investigate the cross‐sectional atomic structure at the homojunction boundaries. As displayed in Figure 2d,e, the sharp and clean atomic structure in each layer can be clearly identified. Meanwhile, disordered atomic arrangements are only observed in a small range at the boundaries, providing intuitive evidence for the existence of pristine van der Waals homogeneous junctions. Figure 2f presents the line profile extracted from the blue rectangle region in Figure 2e, and the distance between atomic layers in 2H‐MoTe_2_ is calculated to be 0.68 nm. Additionally, Figure 2g,h presents the EDS mapping of the 2H‐MoTe_2_ homojunction, revealing the uniform distribution of telluride, molybdenum, and platinum elements. These results unambiguously demonstrate the formation of pristine 2H‐MoTe_2_ homojunction, which holds great potentials for the fabrication of multifunctional optoelectrical devices.

### 2.3 Layer‐Engineered Energy Bands of 2H‐MoTe_2_

**Figure 3:**
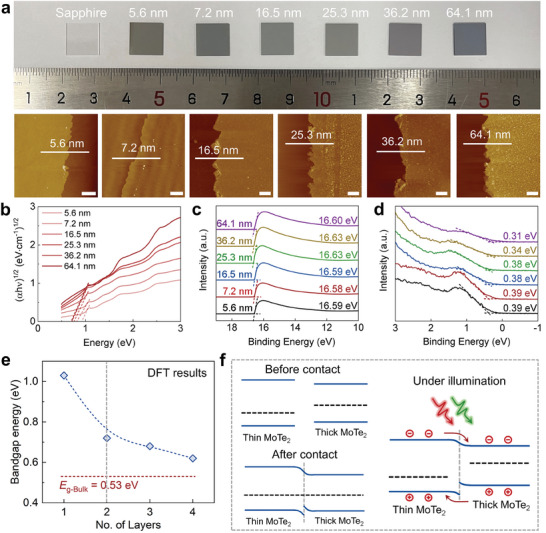
Thickness‐dependent properties of 2H‐MoTe_2_ layers. a) Photograph of the 2H‐MoTe_2_ layers with different thicknesses on sapphire substrates. The middle images are the corresponding AFM images with the scale bars of 2 µm. b) The Tauc's plot of (*αhv*)_1/2_ versus *E*_g_ of 2H‐MoTe_2_ layers with different thicknesses. c) Second electron cut‐off regions and d) the energy difference between the Fermi level (*E*_F_) and valance band maximum (VBM) extracted by ultraviolet photoemission spectroscopy (UPS) analysis. e) Plot of energy band gaps (*E*_g_) versus the number of layers of MoTe_2_ based on DFT calculation results, indicating the decrease in the bandgap energy by increasing the number of layers. f) Schematic diagram of the energy band structure before and after contact between thin and thick 2H‐MoTe_2_ layers.

The energy band structures of 2D semiconductor materials play a crucial role in determining their electrical and optoelectrical properties.^[^ ^34^ ^]^ To investigate the changes in energy band structures of layer‐engineered 2H‐MoTe_2_ and gain insights into the photodetection mechanism of 2H‐MoTe_2_ homojunctions, we have deposited molybdenum layers with increased thicknesses to synthesize 2H‐MoTe_2_ layers. **Figure** **3**a shows the digital images of 2H‐MoTe_2_ films on sapphire substrates with thickness of 5.6, 7.2, 16.5, 25.3, 36.2, and 64.1 nm. As the thickness increases, the color of the wafer gradually deepens from light to dark gray. The AFM and height profiles depicted in Figure 3a and Figure S4 (Supporting Information) serve as compelling evidence that reveals the thickness of the 2H‐MoTe_2_ layers. Meanwhile, the dominant Raman peak (E_2g_^1^) at 234 cm^−1^ in Figure S5 of the Supporting Information shows a sudden decrease as the thickness increases to 16.5 nm, which is consistent to the reported work.^[^ ^21^ ^]^

Furthermore, the changes in energy band structures of 2H‐MoTe_2_ with varying thicknesses are systematically investigated using both UV–vis absorption spectroscopy and ultraviolet photoemission spectroscopy (UPS).^[^ ^35^ ^]^ Figure S6a of the Supporting Information illustrates the UV–vis spectra of 2H‐MoTe_2_ samples and Figure 3b shows the corresponding Tauc's plots extracted from Figure S6a of the Supporting Information with increasing thicknesses. The intensity of light absorption gradually intensifies, and the bandgap obtained from Tauc's plots decreases. The bandgaps of 2H‐MoTe_2_ exhibit a reduction from 0.88 to 0.7 eV as the thickness increases from 5.6 to 36.2 nm. Moreover, the bandgap remains stable at 0.7 eV when the thickness exceeds 36.2 nm, as depicted in Figure S6b of the Supporting Information. Additionally, UPS characterizations are employed to illustrate the second electron cut‐off regions and the energy difference between the Fermi level and valence band in 2H‐MoTe_2_ layers. These results are shown in Figure 3c,d. Combining the results from UV–vis and UPS, the work function (*W*_F_), valance band maximum (VBM), and conduction band minimum (CBM) values of layer‐engineered 2H‐MoTe_2_ are obtained. As shown in Figure S7 of the Supporting Information, the *W*_F_ decreases from 4.63 to 4.59 eV, VBM values increase from −5.02 to −4.93 eV, and CBM values decrease from −4.14 to −4.23 eV as the thickness increases from 5.6 to 64.1 nm. And it is worth noting that the energy band structure remains similar when the thickness exceeds 36.2 nm.

In order to gain a deeper understanding of the variation in energy band of 2H‐MoTe_2_ with different layers, density functional theory (DFT) calculations are performed to simulate the relaxed structures, energy bands, and density of states (DOS).^[^ ^28^, ^36^ ^]^ Detailed simulation results are illustrated in Figures S8–S10 of the Supporting Information. Figure S8a of the Supporting Information shows the lateral and top views of the relaxed structure of 2H‐MoTe_2_ in Figure S8a of the Supporting Information, while Figure S8b of the Supporting Information displays the top and side views of charge density for 2H‐MoTe_2_. These results reveal that the electron density of the telluride atom, which has a higher electronegativity than that of molybdenum atom. Besides, the layer‐engineered band structures and DOS of 2H‐MoTe_2_ have been meticulously analyzed to uncover the connection between electronic properties and the MoTe_2_ with a single layer, 2, 3, 4 layers and bulk structure, which are shown in Figures S9–S10 of the Supporting Information. As the layers of 2H‐MoTe_2_ increases, the bandgap decreases until approaches the bandgap value of bulk MoTe_2_. This observation aligns with previous studies. Figure 3e summarizes this relationship between bandgaps and the layer numbers of MoTe_2_. The relationship between the energy band and thickness of 2H‐MoTe_2_ is consistent in the theoretical and experimental results. However, there exist discrepancies in the precise thickness values, which can be attributed to the simulated environment in a vacuum at absolute zero centigrade, as well as the presence of defects in the synthesized 2H‐MoTe_2_ layers.

Through the layer‐dependent energy bands of 2H‐MoTe_2_, a self‐powered photodetector is achieved by creating a 2H‐MoTe_2_ homojunction with asymmetric thickness. Figure 3f illustrates the band diagrams of the homojunction both before and after contact. The presence of a higher Fermi level in the thick region compared to the thin region leads to the transfer of electrons in Fermi level from the thick layer to the thin layer upon contact, establishing an equilibrium state. This process creates a built‐in electric field at the boundary, with the electric field direction directing from the thick region to the thin region. As a result, the energy bands of the thick and thin regions bend upward and downward, respectively. When the homojunction is illuminated with light, electrons in the valence energy band are excited to the conduction energy band, generating electron–hole pairs. Then the photogenerated electrons migrate from thin sections to thick section and photogenerated holes transfer from thick section to thin section under the influence of the built‐in electric field. This migration of carriers results in the generation of photocurrent. Remarkably, the self‐powered photodetection of the 2H‐MoTe_2_ homojunction is achieved under both visible and near‐infrared light illumination.

### 2.4 Self‐Powered Photodetection of 2H‐MoTe_2_ Homojunctions with Thickness Difference Increasing

**Figure 4:**
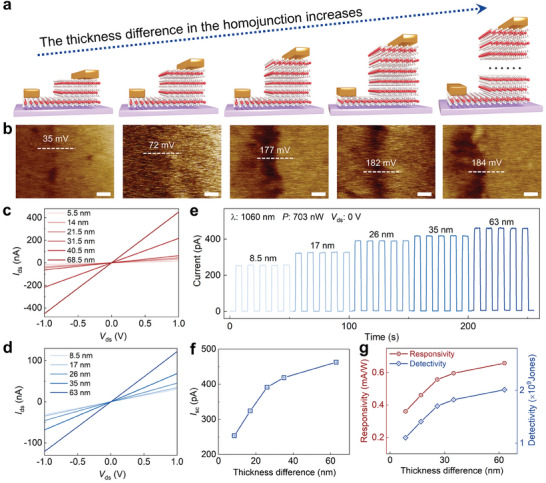
2H‐MoTe_2_ homojunctions with thickness difference increasing. a) Schematic diagram of the 2H‐MoTe_2_ homojunction device with thickness difference between thick region and thin region increasing. b) The Kelvin probe force microscopy (KPFM) mapping of the 2H‐MoTe_2_ homojunction with thickness difference increasing. The scale bar is 1 nm. c) Output curves of 2H‐MoTe_2_ devices with channel thickness increasing from 5.5 to 68.5 nm under dark condition. d) Output curves of 2H‐MoTe_2_ homojunction devices with thickness difference increasing from 8.5 to 63 nm under dark condition. e) Photocurrent response of 2H‐MoTe_2_ homojunction devices with thickness difference increasing. f) Short‐circuit current as a function of thickness difference in the 2H‐MoTe_2_ homojunction under 1060 nm illumination with 703 nW light power. g) The corresponding light power dependent responsivity and detectivity of the device, respectively.

As the Fermi level of 2H‐MoTe_2_ gradually increases with increasing thickness within a certain range, the built‐in electric field of the 2H‐MoTe_2_ homojunction with asymmetric thickness will also be impacted. To comprehensively explore the self‐powered photodetection of the 2H‐MoTe_2_ homojunctions influenced by the thickness difference in the homojunction, a series of two‐terminal devices have been fabricated. The thickness of the thin 2H‐MoTe_2_ region is maintained at a constant 5.5 nm. Meanwhile, the thickness difference between the thick region and the thin region is increased from 8.5 up to 63 nm. **Figure** **4**a shows the corresponding schematic diagrams of the 2H‐MoTe_2_ homojunction device. To ensure the successful formation and modulation of the built‐in electric field at the homojunction boundaries with thickness difference increasing, Kelvin probe force microscopy is adopted to characterize the surface potential difference between thin and thick 2H‐MoTe_2_ layers.^[^ ^37^, ^38^, ^39^ ^]^ Figure 4b displays the surface potential mapping images of the 2H‐MoTe_2_ homojunction. And the corresponding surface potential profiles are shown in Figure S11 of the Supporting Information. As the thickness difference increases from 8.5 to 63 nm, the surface potential difference in the homojunction gradually rises from 35 to 184 mV, which means that the built‐in electric field is enhanced by increasing the thickness difference of the 2H‐MoTe_2_ homojunction. However, when the thickness difference exceeds 26 nm, the surface potential difference of the homojunctions becomes similar, which correlates with the changes in Fermi level as the 2H‐MoTe_2_ thickness increases.

Moreover, the output curves of the 2H‐MoTe_2_ layers with increased thickness are tested and shown in Figure 4c. As the 2H‐MoTe_2_ thickness increases, the current at a constant voltage increases, which suggests the enhancement of conductivity for the lateral 2H‐MoTe_2_ device. The output curves of 2H‐MoTe_2_ homojunctions are shown in Figure 4d. The conductivity of the 2H‐MoTe_2_ homojunction is also enhanced by increasing the thickness difference. Figure 4e displays the photoresponse of the 2H‐MoTe_2_ homojunction device with increased thickness difference under 1060 nm illumination with 703 nW at zero bias. The obvious photoresponse at zero bias demonstrates that this 2H‐MoTe_2_ homojunction device has self‐powered photodetection. Moreover, the photoresponse is enhanced under the synergistic effect of the enhancement of the built‐in electric field and conductivity with increasing thickness. The photocurrent is defined as the difference between the current in the presence of light and in the dark condition at zero bias, which is equal to the short‐circuit current (*I*_sc_) to evaluate the photoresponse capability of self‐powered photodetector. Consequently, the thickness difference in the 2H‐MoTe_2_ homojunction increasing from 8.5 to 63 nm results in an increase in the short‐circuit current from 255 to 460 pA, which is shown in Figure 4f.

Figure 4g displays the thickness difference‐dependent responsivity and detectivity, respectively, which are extracted from the data shown in Figure 4e. The responsivity (*R*) of the 2H‐MoTe_2_ homojunction‐based photodetector reflects the ability to convert incident light into an electrical signal. The *R* value is calculated by *R* = *I*_sc_/*P*, where *P* is the effective light power at a wavelength of 1060 nm. Additionally, the sensitivity of a self‐powered photodetector, which represents the ability to detect the smallest detectable signal, is another important figure of merit. It can be quantified using the detectivity (*D* ^*^) with the following formula^[^ ^6^ ^]^

(1) $$D^{*}=\frac{\sqrt{AB}}{\mathrm{NEP}}\;\approx \frac{R}{\sqrt{\frac{2qI_{d}}{A}}}$$

where *A* and *I*_d_ are the effective area and current under dark condition, respectively. Due to the positive effect of the increase in thickness difference for the 2H‐MoTe_2_ homojunction on the photoresponse under zero bias, both the responsivity and detectivity are enhanced with the thickness difference increasing.

### 2.5 Self‐Powered Photodetection Properties Based on 2H‐MoTe_2_ Homojunctions

**Figure 5:**
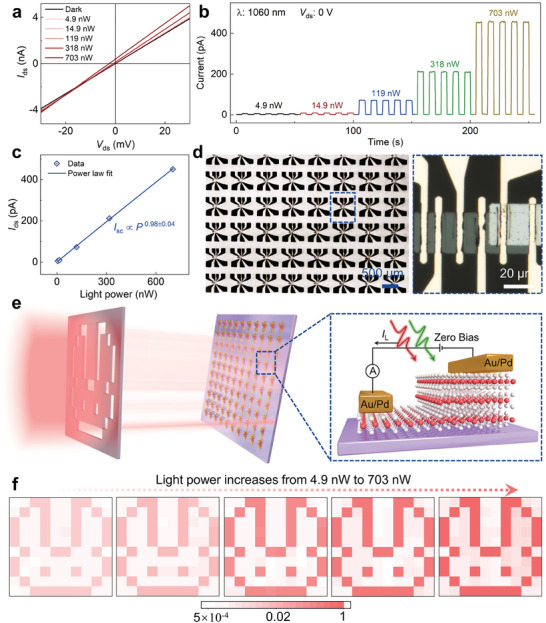
Self‐powered imaging functions of integrated 2H‐MoTe_2_ homojunctions. a) Output curves of the device in the dark and under 1060 nm light illumination with different light intensities. b) Photocurrent response of the 2H‐MoTe_2_ homojunction under 1060 nm with increased light intensities at zero bias. c) Short‐circuit current as a function of light intensities of the 1060 nm light. d) The digital image of the 2H‐MoTe_2_ homojunction devices array and the corresponding optical image of the single device with thickness difference of 63 nm. e) Schematic diagram of imaging process based on the 2H‐MoTe_2_ homojunction devices array and right image is the detailed structure of 2H‐MoTe_2_ homojunction device with asymmetric thickness. f) The imaging results under the wavelength of 1060 nm with light power of 4.9, 14.9, 119, 318, and 703 nW at zero bias.

To comprehensively explore the self‐powered photodetection, a two‐terminal device based on the 2H‐MoTe_2_ homojunctions with thickness difference of 63 nm has been fabricated and detected under near‐infrared and visible illumination. **Figure** **5**a illustrates the output curves of the 2H‐MoTe_2_ homojunction device under 1060 nm illumination. As the light power is gradually enhanced from 4.9 to 703 nW, the photogenerated carriers are increased, resulting in an increase in the short‐circuit current from 5.5 to 460 pA. And the output curve under dark condition passes through the zero‐point, which is due to there is no net current in the material under the dynamic equilibrium between the diffusion and drift of carriers. Figure 5b shows the photoswitching behaviors under different light power of the 2H‐MoTe_2_ homojunction device. The photocurrent increases significantly with the enhancement of light power under 1060 nm illumination. This can be attributed to more photogenerated carriers in the homojunction device under higher light power. Figure 5c shows the light power‐dependent photocurrent, which are extracted from the data shown in Figure 5b. The photocurrent exhibits a nearly positive correlation with the light power. The fitted power law for the relationship between the *I*_sc_ and the *P* at a wavelength of 1060 nm is *I*_sc_ ∝ *P* ^0.98 ± 0.04^. The exponent in the formula, slightly less than 1, is indicative of the complex photoexcitation process in the 2H‐MoTe_2_ homojunction‐based photodetector. This process includes the generation, recombination, and trapping of electron–hole pairs, which lead to a deviation from a simple linear relationship between *I*_sc_ and *P*.^[^ ^8^, ^40^ ^]^ Moreover, the exponent value close to 1 signifies the high quality of the 2H‐MoTe_2_ homojunction, indicating efficient photoresponse characteristics and a well‐optimized performance.

Additionally, the responsivity and detectivity shown in Figure S12 of the Supporting Information decrease with the increased light power and remains relatively stable with values of 0.63 ± 0.03 mA W^−1^ and 1.38 ± 0.05 × 10^9^ cm Hz^1/2^W^−1^ as the light power increases more than 14.9 nW. The consistent *R* and *D* ^*^ across different light power indicate that the photoelectric conversion efficiency and sensitivity of the photodetector is not significantly influenced by light power. Furthermore, to assess the response and recovery speeds of the 2H‐MoTe_2_ homojunction photodetector, a single photoswitching cycle is examined using a high time resolution, as shown in Figure S13 of the Supporting Information. The rising time is 15 ms, which is calculated as the time for the current to rise from 10% to 90% of its maximum value. Similarly, the falling time is calculated to be 17 ms.

The stability and reliability of photoswitching are crucial factors for assessing the potential of 2D materials‐based photodetectors in practical application.^[^ ^7^ ^]^ Figure S14 of the Supporting Information presents the results of the photoswitching process of the self‐powered photodetector based on 2H‐MoTe_2_ homojunction. This result involves hundreds of cycles, with each cycle consisting of a 5 s on‐state and a 5 s off‐state for 5 s under 1060 nm illumination. Notably, there is no obvious deviation in the photocurrent under light illumination, demonstrating excellent stability and reliability of the self‐powered photodetector. However, it is worth noting that there are slight differences in the largest currents after switching the light illumination in these cycles. This is attributed to the inevitably weak fluctuations in the light power of the light source.

The photoresponse of the 2H‐MoTe_2_ homojunction under visible illumination has been investigated. Figure S15a of the Supporting Information illustrates the output curves of the device under both dark condition and 520 nm illumination. The higher light power at zero bias leads to the generation of more electron–hole pairs. Consequently, the short‐circuit current is enhanced, as shown in Figure S15b of the Supporting Information. This indicates that the performance of the photodetector improves with light power increasing. Additionally, Figure S15c of the Supporting Information displays the photoresponse results of five switch cycles conducted at varying light power. These results further demonstrate that the short‐circuit current is enhanced by increasing the light power of 520 nm. Moreover, Figure S16 of the Supporting Information illustrates the responsivity and detectivity of the device under 520 nm illumination. Remarkably, both the responsivity and detectivity remain nearly unchanged values of 0.52 ± 0.03 mA W^−1^ and 1.13 ± 0.06 × 10^9^ cm Hz^1/2^W^−1^ with the light power increasing higher than 149 nW at zero bias. Figure S17 of the Supporting Information presents the photoresponse of the device over hundreds of cycles in a long‐time range under 520 nm illumination at zero bias. The 2H‐MoTe_2_ homojunction device exhibits excellent stability and reversible response. These photoresponse characteristics highlight the stable and self‐powered photodetection capability of the 2H‐MoTe_2_ homojunction‐based device in the near‐infrared and visible range. It shows great potential for optoelectronics application with low consumption and a wide photodetection range.

### 2.6 Integrated 2H‐MoTe_2_ Homojunctions for Self‐Powered Imaging

Motivated by the fabrication process of patterned 2H‐MoTe_2_ homojunctions and excellent self‐powered photodetection in the near‐infrared range, an image sensor has been developed. This image sensor comprises a grid of 10 × 10 individual 2H‐MoTe_2_ homojunction devices with thickness difference of 17 nm. Figure 5d displays the corresponding optical images of the fabricated devices array and the single device. The image reveals the constructed 10 × 10 2H‐MoTe_2_ homojunctions, which appear as the gray regions. Additionally, bright yellow part in the optical image represents the deposited 50 nm/10 nm Au/Pd electrodes and the channel area measures 30 × 10 µm^2^. Figure 5e illustrates the schematic diagram of the imaging process. In this setup, near‐infrared light is directed through a “rabbit” mask to selectively illuminate the array of 2H‐MoTe_2_ homojunction devices. The right section in Figure 5e provides a detailed view of the single device structure, revealing that each pixel within this array is composed of a layer‐engineered 2H‐MoTe_2_ homojunction device.

Upon illuminating the image sensor through the mask at zero bias, the pixels in the dark condition exhibit ultralow current and other pixels under light illumination display short‐circuit current of several hundreds of picoampere, respectively. Figure 5f illustrates the rabbit‐pattern imaging result obtained from the image sensor with progressive improvement in imaging quality as the light power increases. As the light power increases from 4.9 to 703 nW, the imaging results of the “rabbit” shape becomes more distinct. This demonstrates the capability to distinguish between light and dark conditions under near‐infrared environment. The uniformity of devices performance within the array is a crucial factor to evaluate the quality of an image sensor. Figure S20 of the Supporting Information displays the imaging result through an imaging process without a mask. The unified color distribution observed under 1060 nm illumination serves as compelling evidence for the consistency across the entire array, which indicates that the devices exhibit similar characteristics and perform reliably. These imaging results utilizing the 10 × 10 array of 2H‐MoTe_2_ homojunction devices demonstrate promising prospects for the practical applications in the field of ultralow power consumption and infrared imaging.

## 3 Conclusion

In summary, a pristine van der Waals homojunctions based on 2H‐MoTe_2_ layers has been proposed and fabricated by tellurizing molybdenum precursors with asymmetric thickness. This approach enables the creation of high‐quality boundary between thin and thick 2H‐MoTe_2_ layers without lattice mismatch, strain, and defects. Moreover, the layer‐engineered energy bands of the 2H‐MoTe_2_ layers induce difference in Fermi levels at the homojunction boundary, facilitating the formation of built‐in electric fields. This enables the fabrication of self‐powered photodetectors capable of detecting light in the visible and near‐infrared range. Furthermore, imaging functions under near‐infrared illumination are achieved by constructing a 10 × 10 array of 2H‐MoTe_2_ homojunction devices. This work introduces a universal fabrication method for patterned 2D homojunctions with asymmetry thickness, paving the way for integrated photodetectors based on 2D materials with low power consumption.

## 4 Experimental Section

### Material Synthesis

The synthesis of 2H‐MoTe_2_ layers on sapphire substrates (1 cm × 1 cm) was carried out by using a CVD method. To create patterned Mo layers with varying thicknesses, a combination of standard photolithography and magnetron sputtering techniques was employed. The thickness of the patterned Mo layer was controlled by adjusting the sputtering time, ranging from 15 to 120 s. Subsequently, a thermal‐assisted tellurization process was utilized to synthesize 2H‐MoTe_2_ homojunctions with asymmetric thickness. This involved the use of 1.0 g high purity Te powder (99.99%, Aladdin) placed upstream of the quartz tube, along with the Mo layer precursors at the heating center. During this tellurization process, the system was heated to 660 °C within 20 min and maintained for 150 min, using a mixed flux of H_2_ (5 sccm) and Ar (4 sccm). After the tellurization process, the system was cooled to room temperature using a high flux of Ar (200 sccm).

### Characterization

The shapes and sizes of the Mo layer precursor, 2H‐MoTe_2_ homojunctions, and the 2H‐MoTe_2_ device were characterized using an optical microscope (LEICA DMLM). The thickness of the 2H‐MoTe_2_ layers was determined by an AFM (Dimension Icon, Bruker). Raman spectra and the Raman mapping were obtained using a Renishaw Raman RM3000 scope with the 514 nm excitation from an Argon laser. Ultraviolet photoelectron spectroscopy (UPS) analysis was performed by a PHI 5600 (Physical Electronics) instrument. The UV–vis absorption spectroscopy of the layer‐dependent 2H‐MoTe_2_ layers was conducted using a Lambda 1050+ spectrophotometer (PerkinElmer). For the synthesized MoTe_2_ films on sapphire substrates, the polymethylmethacrylate layers were coated onto the MoTe_2_ film. These films were exfoliated from the substrates onto a copper grid for the transmission electron microscopy (TEM), scanning mode TEM (STEM), SAED, and energy dispersive spectrometer (EDS) characterizations, which were carried out using a JEM 2010F (JEOL) instrument.

### Device Fabrication and Measurement

The fabrication of the 2H‐MoTe_2_ homojunction devices array involved standard photolithography and electron beam evaporation to deposit a 30 nm thick layer of Au onto the 2H‐MoTe_2_ array. The optoelectronic characterization of the 2H‐MoTe_2_ homojunction device was performed using a combination of a Keithley 4200 SCS semiconductor parameter analyzer and a probe station (FormFactor MPS 150, Livermore, CA, USA). Illumination of the device was realized by light with wavelengths of 520 nm (MW‐GX‐520/700 mW) and 1060 nm (MW‐GX‐1060/1000 mW). The corresponding light intensities were measured through a power meter (Vega Color Laser Power & Energy Meter). Each individual device within the 2H‐MoTe_2_ homojunction devices array was illuminated and measured sequentially. All measurements were conducted at atmospheric pressure and room temperature. The pixel of the 10 × 10 self‐powered photodetectors array was illuminated and measured one by one, and the pixel current was received sequentially. The light source was placed above the device with distance of 20 cm.

### Theoretical Calculation

The energy bands and structures of 2H‐MoTe_2_ with different layers were simulated using DFT. The Perdew–Burke–Emzerhof was employed, along with plane‐wave cutoff energy of a 520 eV. For structural relaxation, a 6 × 6 × 1 k‐point grid was utilized in the Brillouin zone, following the Gamma‐point scheme. To avoid interactions between periodic structures in the simulation of thin layered materials, a vacuum space of 20 Å was included in a 1 × 1 supercell of 2H‐MoTe_2_ (with dimensions of 3.57 Å × 3.57 Å), which was shown in Figure S8a of the Supporting Information. The convergence criteria for the simulation were set to 10^−6^ eV for energy and 0.025 eV Å^−1^ for atomic forces. Moreover, DFT‐D3 method was employed to account for van der Waals structure of 2H‐MoTe_2_. For the calculation of the DOS and energy bands, a 12 × 12 × 1 k‐point grid was used for the Brillouin zone, and a convergence criteria of 10^−7^ eV was applied.

## Conflict of Interest

The authors declare no conflict of interest.

## Supporting information

Supporting Information

## Data Availability

Research data are not shared.
