# Supplementary material for: Integrated Pristine van der Waals Homojunctions for Self‐Powered Image Sensors
Source: Adv Mater. 2024 Jul 18;37(23):2404013. doi: 10.1002/adma.202404013 (PMC12160690; doi:10.1002/adma.202404013)
Supplement: Supplementary file 1 — Supporting Information [file ADMA-37-2404013-s001.docx]

Supporting Information

Integrated pristine van der Waals homojunctions for self-powered image sensors

Yunxia Hu, Jun Wang, Mohsen Tamtaji, Yuan Feng, Tsz Wing Tang, Mohammadreza Amjadian, Ting Kang, Mengyang Xu, Xingyi Shi, Dongxu Zhao, Yongli Mi, Zhengtang Luo*, Liang An*

**Contents:**

**S1. Fabrication of 2H-MoTe_2_ homojunctions with asymmetric thickness.**

**S2. Characterizations of 2H-MoTe_2_ homojunctions.**

**S3. Thickness-dependent energy bands of 2H-MoTe_2_ layers.**

**S4. DFT calculations about layer-dependent energy bands of 2H-MoTe_2_ layers.**

**S5. Surface potential difference of 2H-MoTe_2_ homojunctions.**

**S6. Photodetection properties of 2H-MoTe_2_ homojunctions under 1060 nm.**

**S7. Photodetection properties of 2H-MoTe_2_ homojunctions under 520 nm.**

**S8. Comparison of self-powered photodetection in the 2H-MoTe_2_ homojunction device.**

**S9. Metal-semiconductor contact of the 2H-MoTe_2_ homojunction device.**

**S10. Imaging functions of the 2H-MoTe_2_ homojunctions array under 1060 nm.**

**S1. Fabrication of 2H-MoTe_2_ homojunctions with asymmetric thickness.**


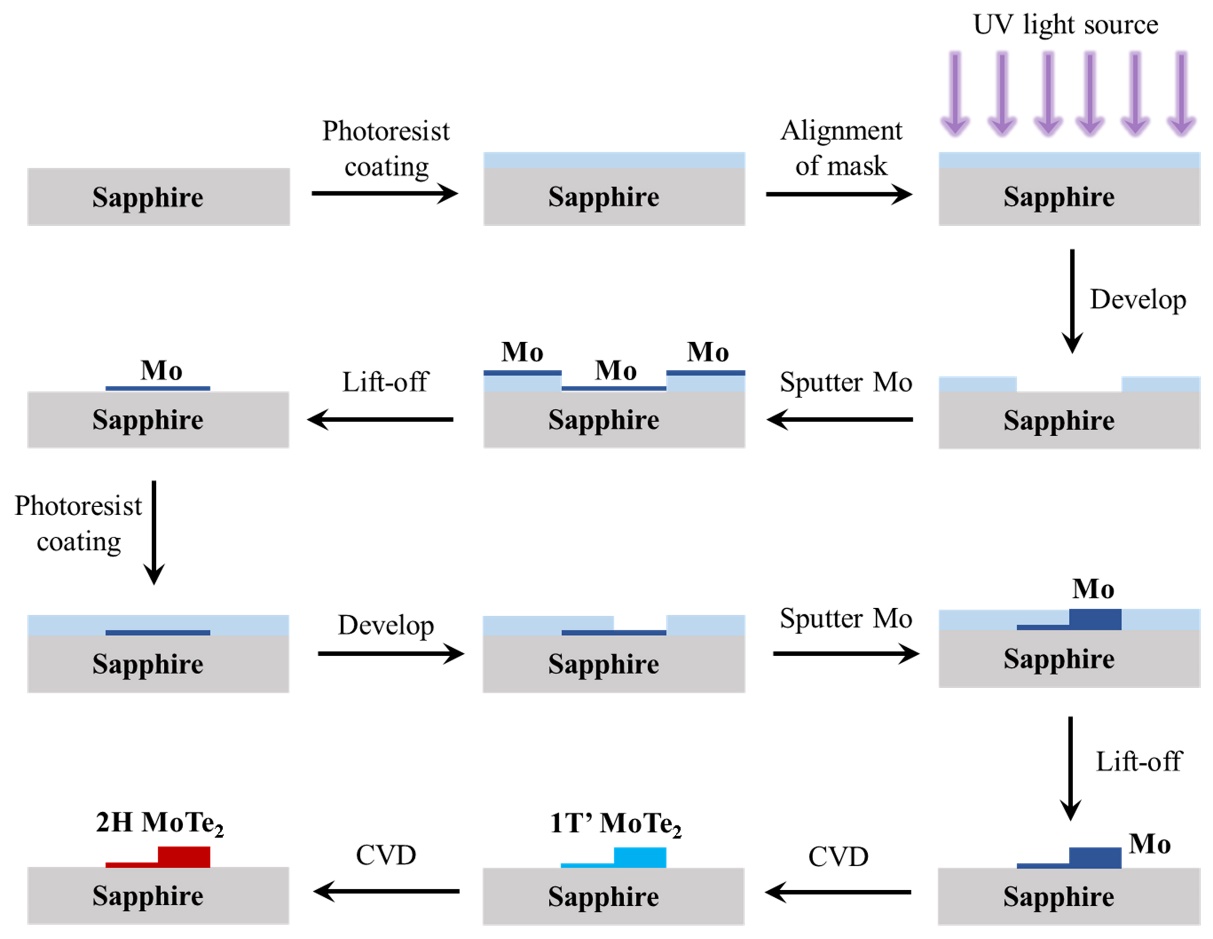


**Figure S1.** Schematic illustrations of the process flow for fabricating the 2H MoTe_2_ homojunction with asymmetric thickness.

**S2. Characterizations of 2H-MoTe_2_ homojunctions.**


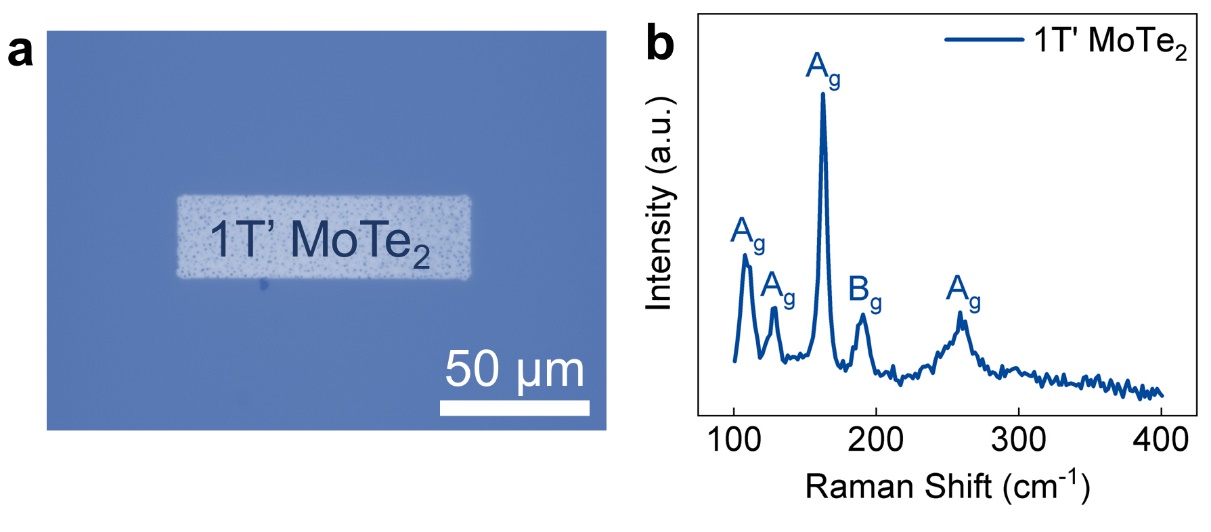


**Figure S2.** a) The optical image of 1T’ MoTe_2_ layer. b) Raman spectra of the 1T’ MoTe_2_ layer.


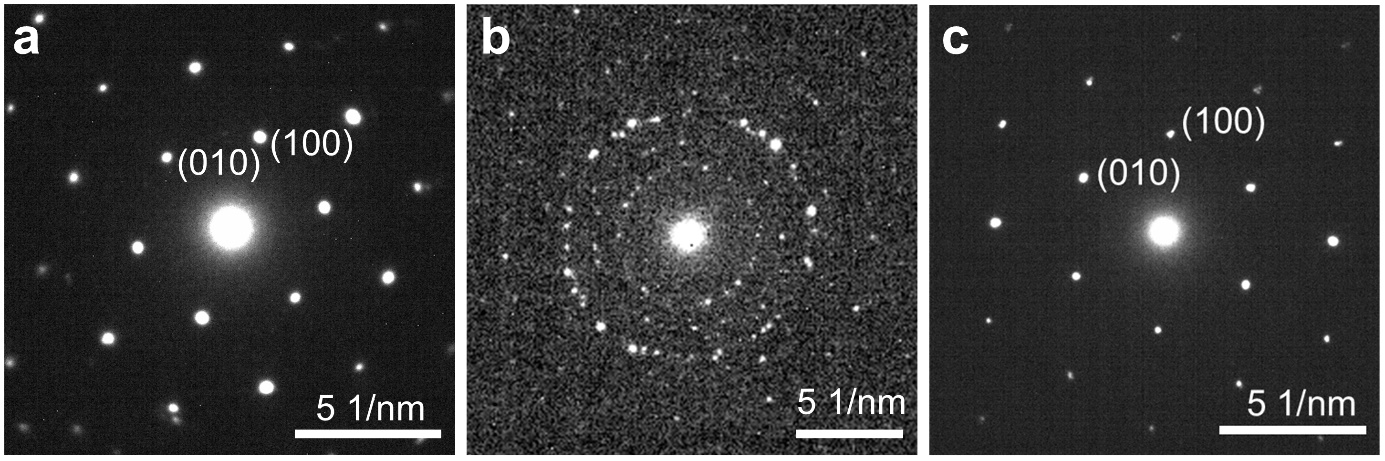


**Figure S3.** SAED images of a) the thick 2H MoTe_2_, b) 2H MoTe_2_ homojunction interface, and c) the thin 2H MoTe_2_ regions, respectively.

**S3. Thickness-dependent energy bands of 2H-MoTe_2_ layers.**


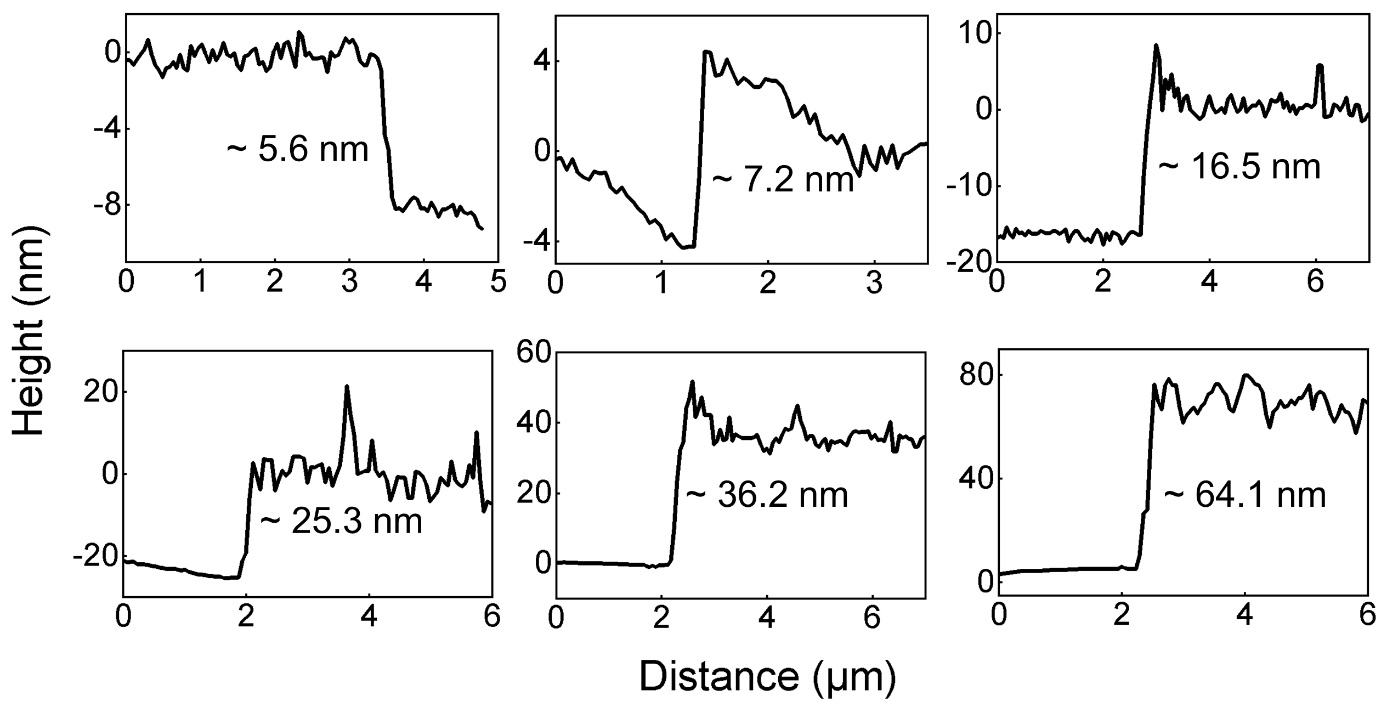


**Figure S4.** The height profiles of the AFM results of 2H MoTe_2_ layers with different thicknesses on sapphire substrates.


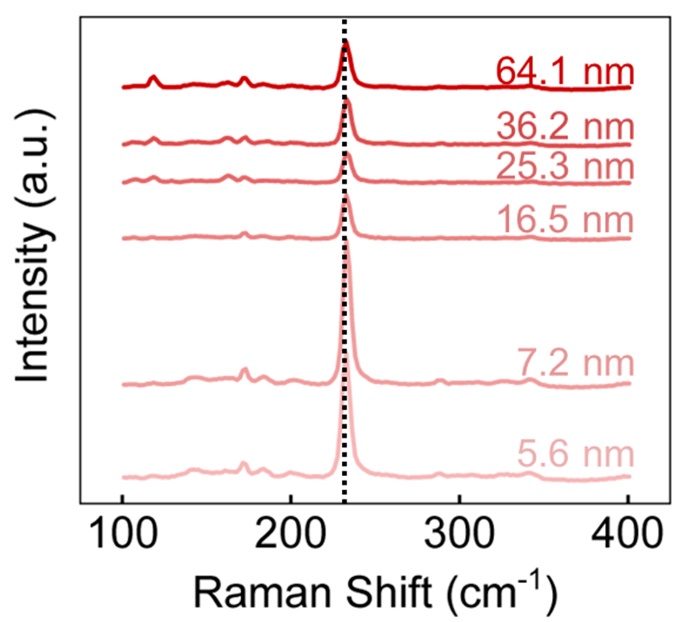


**Figure S5.** The height profiles of the AFM results of 2H MoTe_2_ layers with different thicknesses on sapphire substrates.


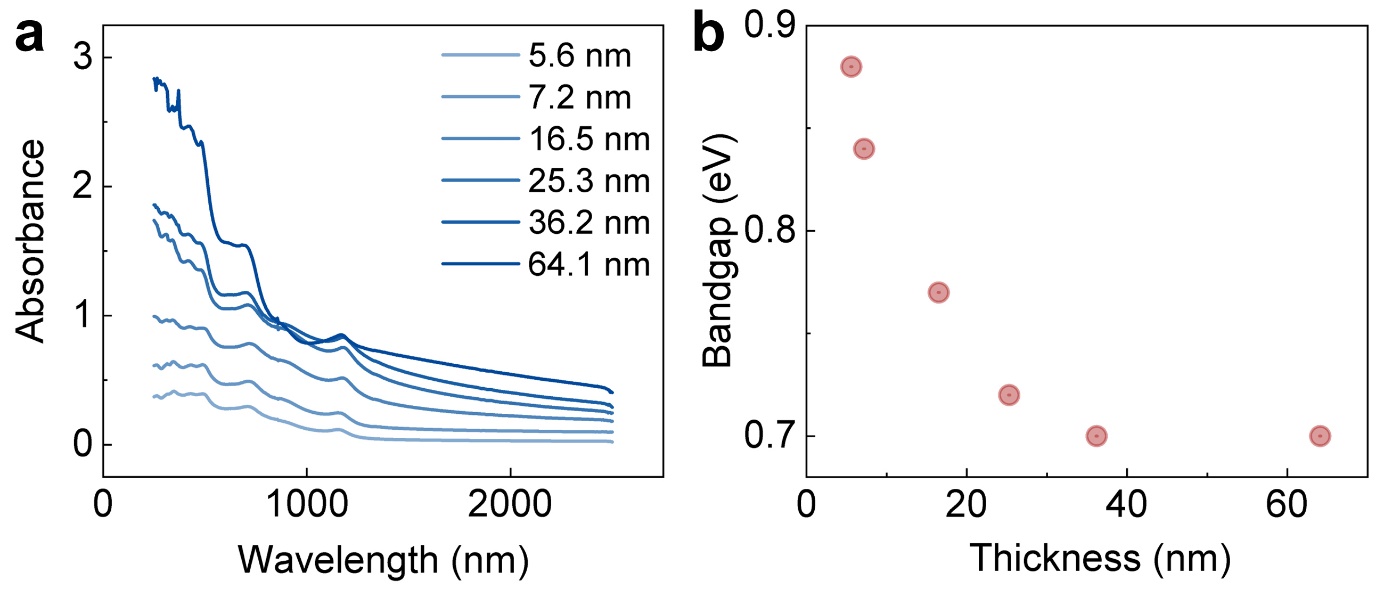


**Figure S6.** a) Optical absorption spectra of 2H MoTe_2_ layers with different thicknesses. b) The optical bandgaps extracted from Tauc plots for 2H MoTe_2_ layers with different layer numbers.


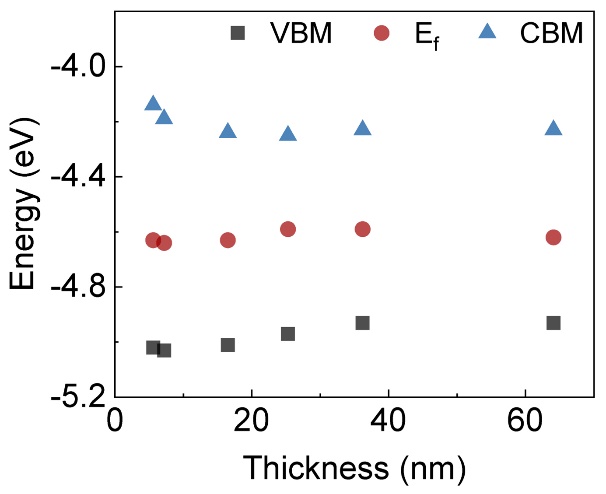


**Figure S7.** The VBM, E_f_, and CBM as a function of thicknesses of 2H MoTe_2_ layers.

**S4. DFT calculations about layer-dependent energy bands of 2H-MoTe_2_ layers.**


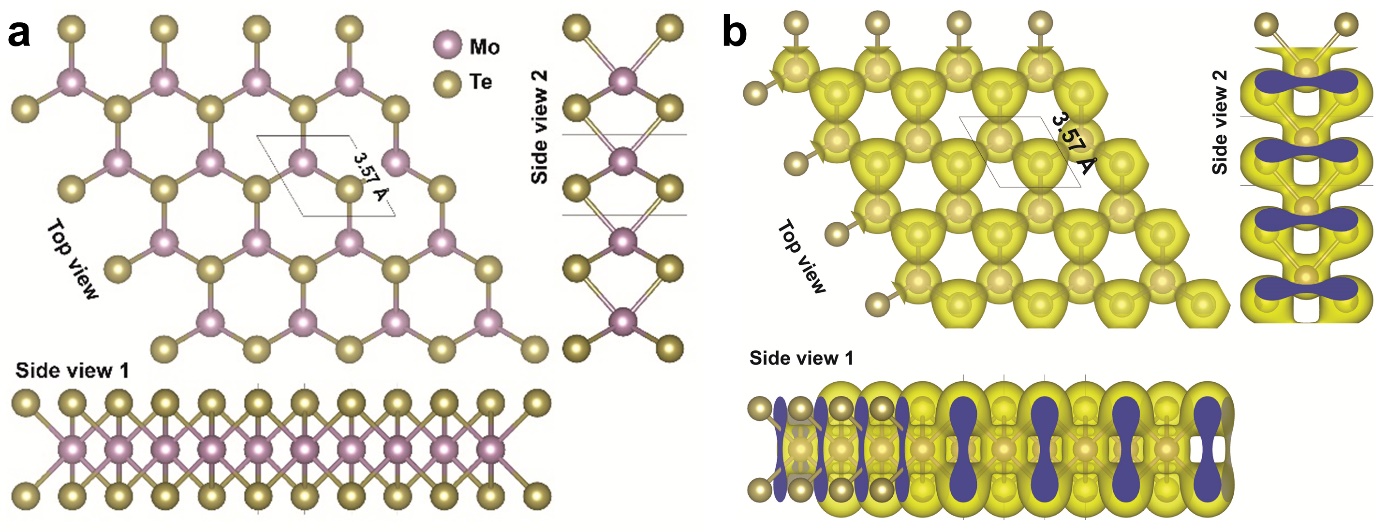


**Figure S8.** a) Lateral view and top views of 1-layer MoTe_2_. Purple and green balls stand for Mo and Te atoms, respectively. b) The top and side views of charge density for MoTe_2_ with the isosurface of 0.06 eÅ^-3^. This indicates that the electron density of Te atom is higher than the electron density of Mo atom due to its higher electronegativity.


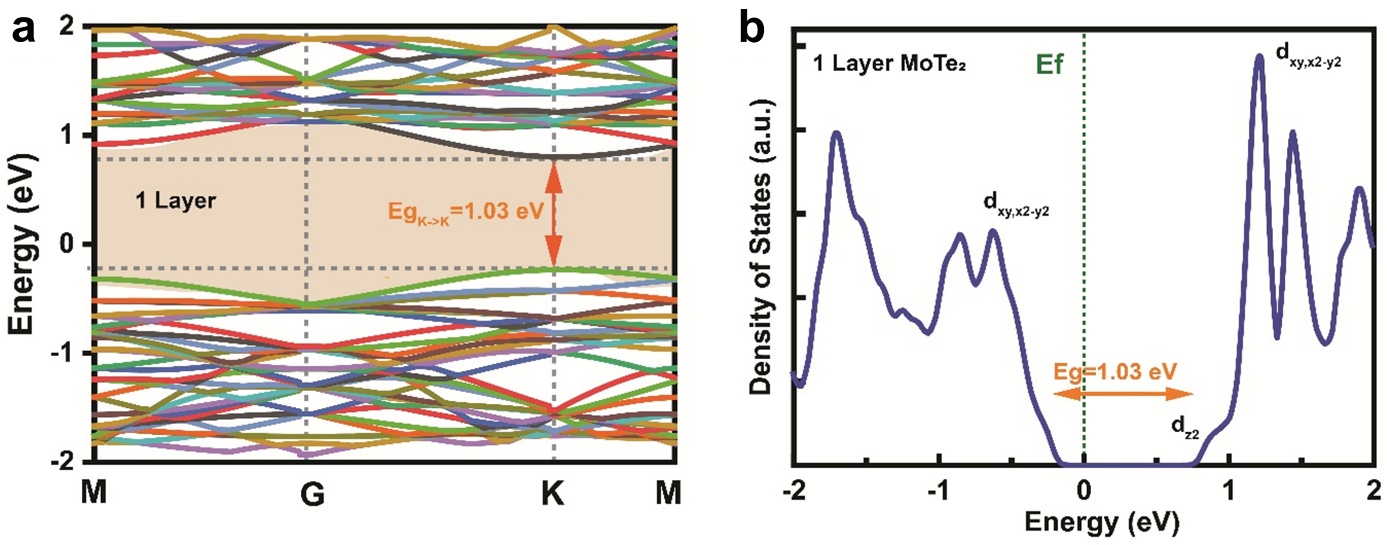


**Figure S9.** a) Band structures and b) density of states (DOS) of the 1-layer MoTe_2_ indicating the K→K direct band structure with 1.03 eV bandgap.


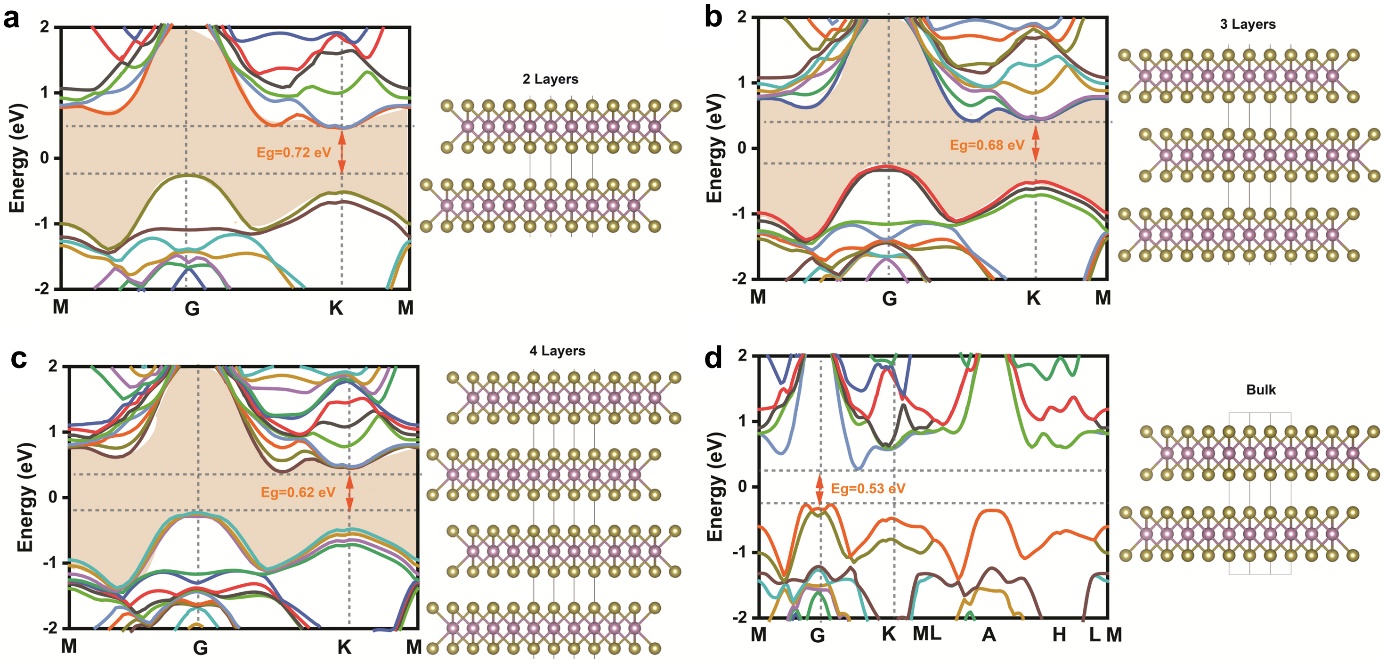


**Figure S10.** Band structures and structure of MoTe_2_ with a) 2 layers, b) 3 layers, and c) 4 layers along with (d) bulk MoTe_2_. This indicates that K→K direct band structure goes to G→K indirect band structure with increasing in the layers.

**S5. Surface potential difference of 2H-MoTe_2_ homojunctions.**


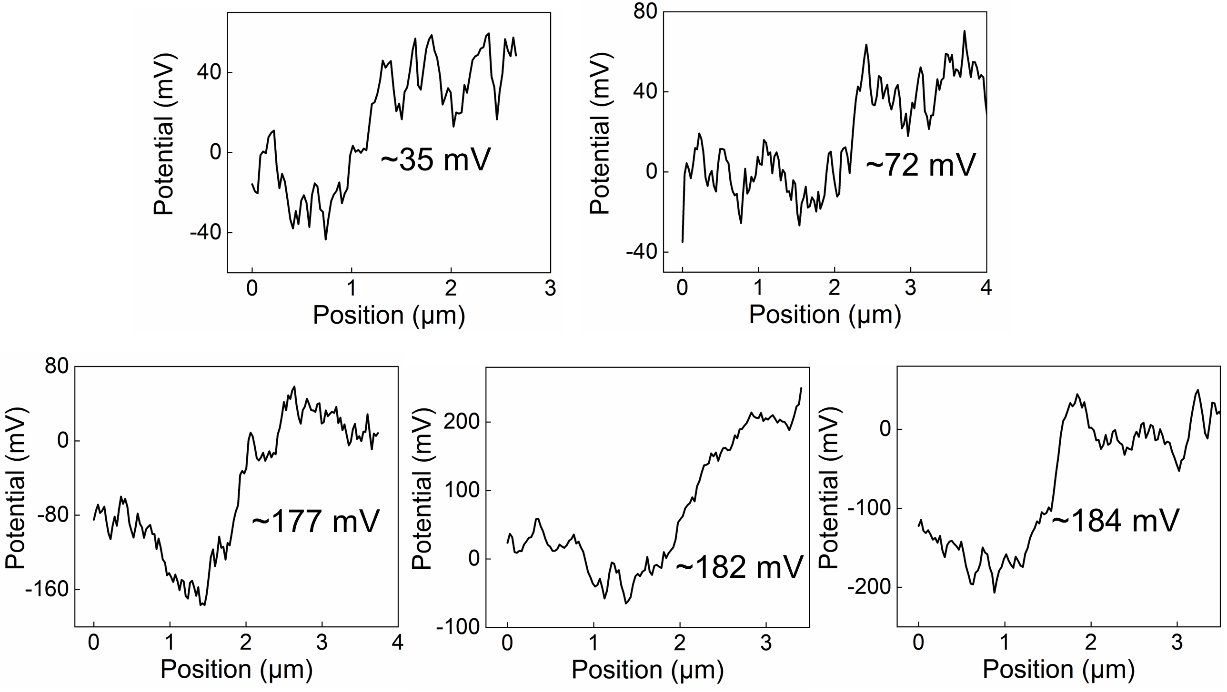


**Figure S11. KPFM characterization results.** Surface potential difference with the thickness difference between 5.5 nm 2H-MoTe_2_ layers and thick 2H-MoTe_2_ of 5.5 nm, 8.5 nm, 16 nm, 26 nm, 35 nm, and 63 nm is a) 35 mV, b) 45 mV, c) 72 mV, d) 177 mV, e) 182 mV, f) 184 mV, respectively.

**S6. Photodetection properties of 2H-MoTe_2_ homojunctions under 1060 nm.**


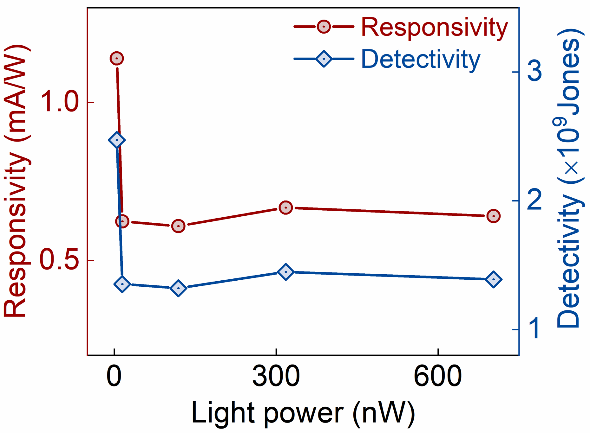


**Figure S12.** The responsivity and detectivity of the 2H-MoTe_2_ homojunction device under increased light intensities with the wavelength of 1060 nm.


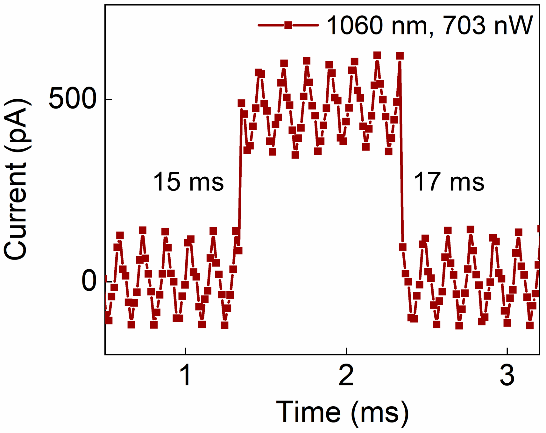


**Figure S13.** The photoresponse of the 2H-MoTe_2_ homojunction device under 1060 nm illumination with light power of 703 nW at zero bias.


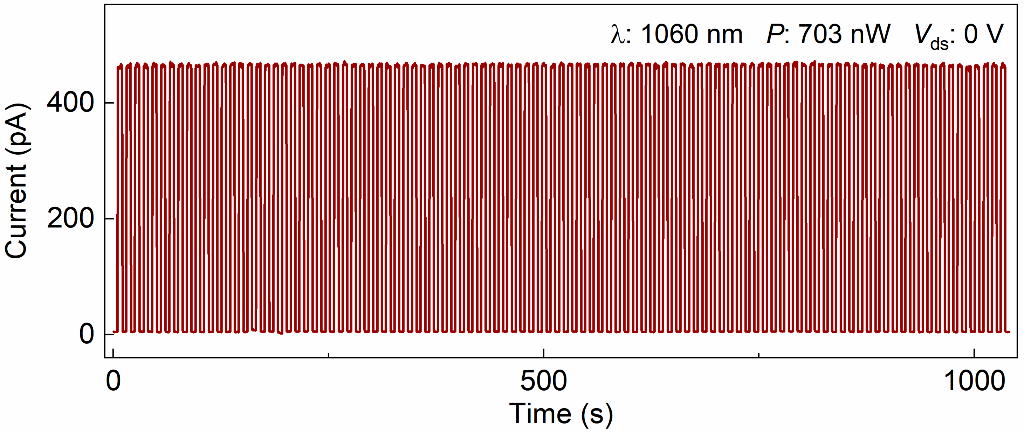


**Figure S14.** Long-time-dependent photoresponse of the 2H-MoTe_2_ homojunction device under 1060 nm illumination with the light power of 703 nW at zero bias.

**S7. Photodetection properties of 2H-MoTe_2_ homojunctions under 520 nm.**


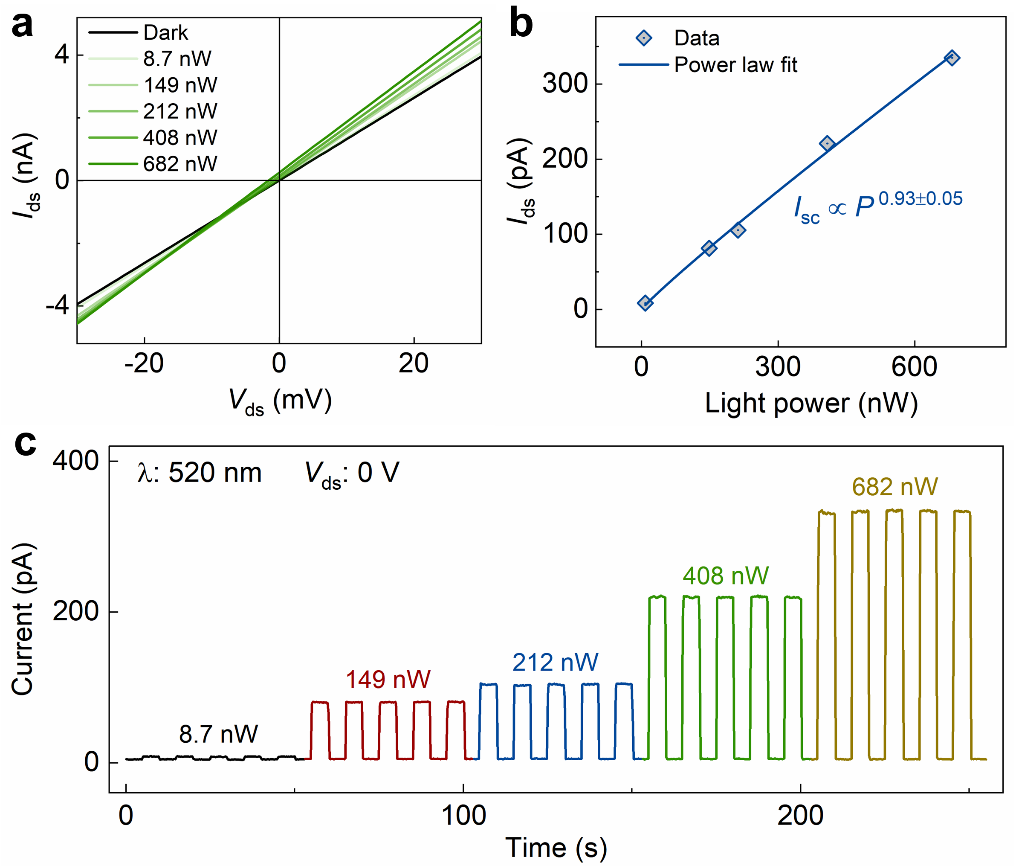


**Figure S15.** a) Output curves of the 2H-MoTe_2_ homojunction with thickness difference of 63 nm in the dark and under 520 nm light illumination with increased light intensities at zero bias. b) Photocurrent as a function of light intensities of the 520 nm light illumination. c) Photoresponse of the device under 520 nm with various light intensities at zero bias.


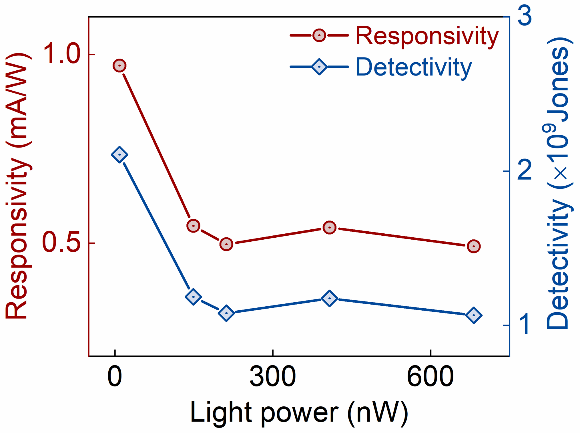


**Figure S16.** The light power dependent responsivity and detectivity of the 2H-MoTe_2_ homojunction device under 520 nm illumination.


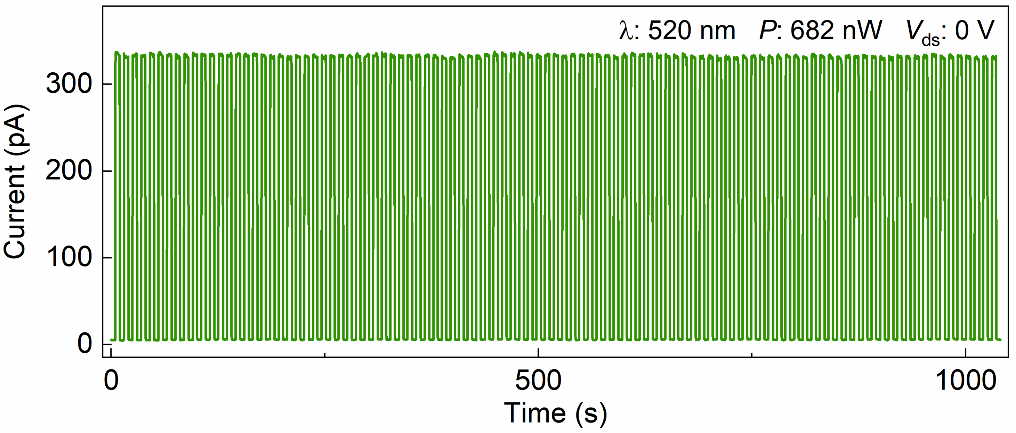


**Figure S17.** Long-time-dependent photoresponse of the 2H-MoTe_2_ homojunction device under 520 nm illumination with the light power of 682 nW at zero bias.

**S8. Comparison of self-powered photodetection in the 2H-MoTe_2_ homojunction device.**

To further analyze the role of homojunctions with asymmetric thickness in the self-powered photodetector, the electric and photoelectric performance of thin 2H-MoTe_2_ region and thick 2H-MoTe_2_ region shown in the optical image of Figure 5d are investigated, respectively. Figure S18a shows the output curves of the thin region, homojunction region, and thick region. The conductivity of thick 2H-MoTe_2_ layers is higher than that of thin 2H-MoTe_2_ layers, resulting in that the current of homojunction is higher than that of thin region and lower than that of thick region under a certain bias. Additionally, without the presence of the built-in electric field in 2H-MoTe_2_ homojunction, self-powered photodetection is predicted to be non-existent. Figure S18b shows the photoresponse of the thin 2H-MoTe_2_ region and thick 2H-MoTe_2_ region under 1060 nm illumination at zero bias. The photocurrents of thin region and thick region are 6.5 pA and 15.5 pA, which is much lower than the photocurrent of 2H-MoTe_2_ homojunction of 460 pA. The photoresponse of 2H-MoTe_2_ layers with uniform thickness may be caused by the asymmetric contact of the electrodes, which has negligible influence on the performance of the 2H-MoTe_2_ homojunction.


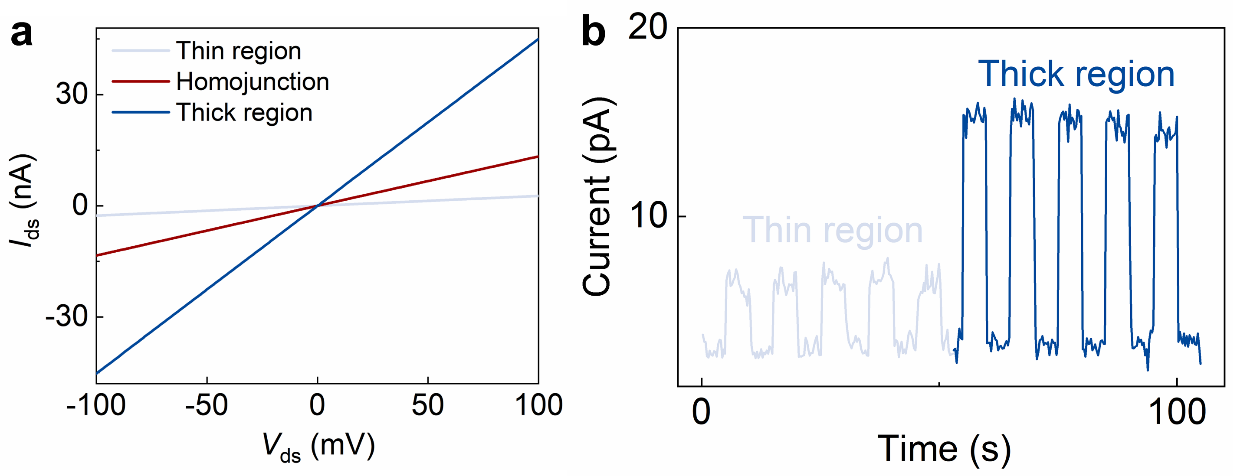


**Figure S18.** a) Output curves of the thin 2H-MoTe_2_ region, 2H-MoTe_2_ homojunction region, and thick 2H-MoTe_2_ region under dark condition. b) The photoresponse of the thin 2H-MoTe_2_ region and thick 2H-MoTe_2_ region under 1060 nm illumination with light power of 703 nW at zero bias.

**S9. Metal-semiconductor contact of the 2H-MoTe_2_ homojunction device.**

To mitigate the impact of the metal-semiconductor Schottky barrier on the self-powered performance of the 2H-MoTe_2_ homojunction devices, the electrodes are selected as the 50 nm/10 nm Au/Pd electrodes. KPFM characterizations are adopted to analyze the difference of surface potentials between metal electrodes and 2H-MoTe_2_ layers. Figures S19a and S19b show the surface potential mapping images with Au and Pd electrodes. And Figures S19b and S19d shows the corresponding potential profiles. It can be seen that the contact barrier between Pd and 2H-MoTe_2_ layers is lower than that between Au and 2H-MoTe_2_ layers, inducing that the photoresponse of 2H-MoTe_2_ homojunction with Pd electrodes is higher than that of 2H-MoTe_2_ homojunction with Au electrodes. Figure S19e shows the photoresponse of 2H-MoTe_2_ homojunction with thickness difference of 17 nm and different electrodes of Au and Pd. The photocurrents of 2H-MoTe_2_ homojunction with Au electrodes and Pd electrodes are 212 pA and 326 pA, which also demonstrate that the contact barrier between Pd and 2H-MoTe_2_ layers is lower than that between Au and 2H-MoTe_2_ layers.


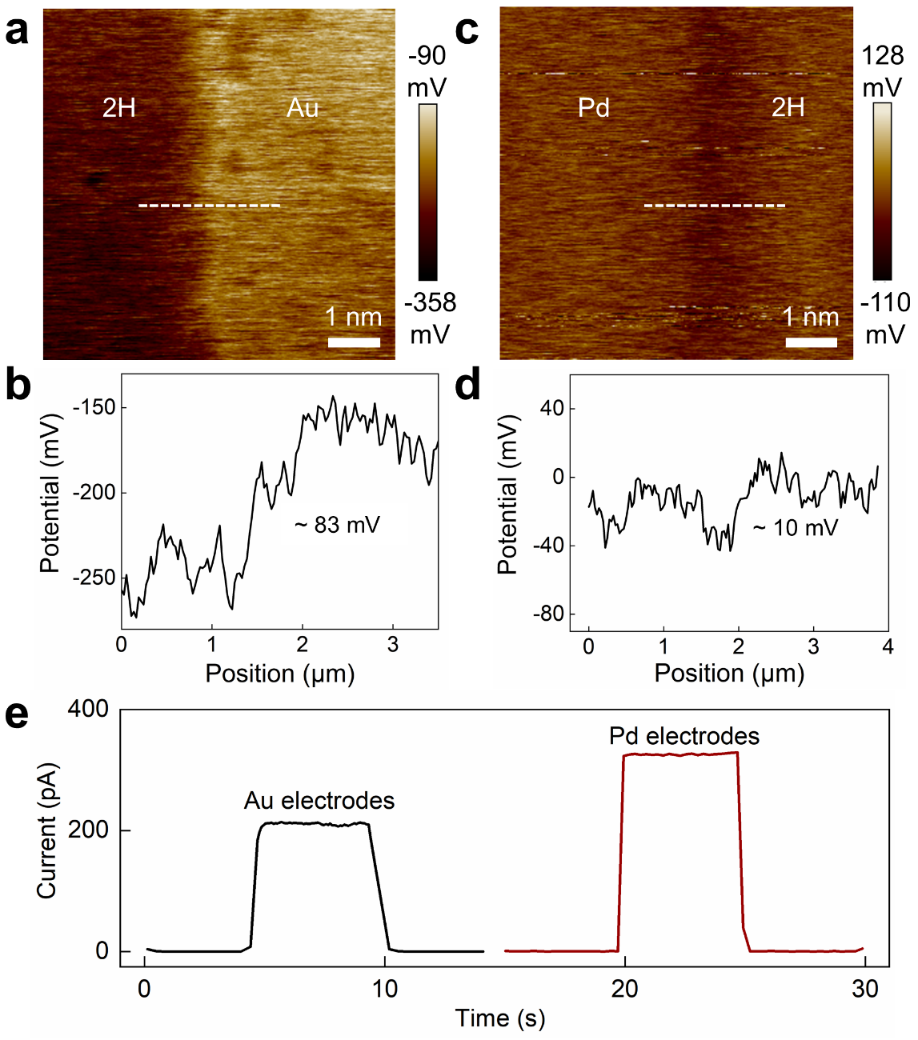


**Figure S19.** a) The KPFM mapping of the interface between 2H-MoTe_2_ and the Au electrode. b) The corresponding potential profile. c) The KPFM mapping of the interface between 2H-MoTe_2_ and the Pd electrode. d) The corresponding potential profile. e) Photocurrent response of 2H-MoTe_2_ homojunction with thickness difference of 17 nm with Au electrodes and Pd electrodes.

**S10. Imaging functions of the 2H-MoTe_2_ homojunctions array under 1060 nm.**


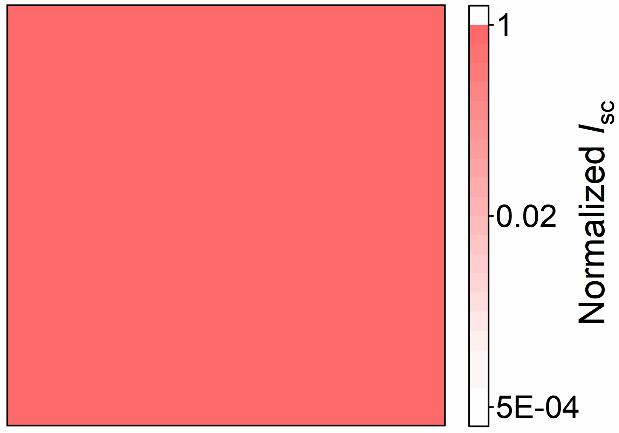


**Figure S20.** The imaging result of the 2H-MoTe_2_ homojunction devices array without applying the mask under 1060 nm illumination with light power of 703 nW at zero bias.
